# How many preterm births in England are due to excision of the cervical transformation zone? Nested case control study

**DOI:** 10.1186/s12884-015-0664-3

**Published:** 2015-09-29

**Authors:** R. Wuntakal, Alejandra Castanon, R. Landy, P. Sasieni

**Affiliations:** Whipps Cross University Hospital, Barts Health NHS Trust, London, England UK; Guys and St Thomas’ Hospital, London, England UK; Centre for Cancer Prevention, Wolfson Institute of Preventive Medicine, Charterhouse Square, London, England EC1M 6BQ UK

**Keywords:** Preterm birth, Cervical treatment, LLETZ, Attributable risk

## Abstract

**Background:**

Preterm births (as a proportion of all births) have been increasing in many countries. There is growing evidence of increased risk of preterm birth following excisional treatment of the cervix. We estimate the number of preterm births attributable to excisional treatments with a length of 10 mm or more in England.

**Methods:**

Case–control study nested in a record linkage cohort of women with a histological sample at 13 hospitals in England. We combined observed age at first excisional treatment in our cohort with the weighted distribution of excision length from the case–control study to estimate the length distribution by age at first treatment among the cohort. The number of births after excision for each 5-year age group was estimated using national fertility data; published absolute risks of preterm (<37 gestational weeks) and very preterm birth (<32 weeks) were applied to these to estimate the number of preterm births per 100 women treated. Excess preterm births were estimated assuming all treatments were small. The attributable risk of preterm birth following excisional treatment in England was estimated.

**Results:**

The majority of first excisional treatments at colposcopy were small (47.5 %) or medium (39.1 %), 9.5 % were large and 4.1 % were very large excisions. 4.0 % of women treated before birth had more than one excisional treatment. Thus based on our cohort of 10,711 treated women and the length of treatment observed in the case control study we estimate an excess of 240 preterm births (including 57 very preterm) or 2.2 (including 0.5 very preterm) per 100 women treated. At a population level (for England) we estimate that 39,101 women aged 20–39 would be treated each year and that these treatments will lead to an excess of 840 preterm births (including 196 very preterm) in England each year.

**Conclusions:**

Assuming associations between preterm birth and treatment for cervical disease are causal; we estimate that an excess 840 (2.5 %) preterm birth in England each year are due to excisional treatments of 10 mm or more. Those that go on to become pregnant should be closely monitored during antenatal period to reduce their risk of preterm birth.

**Electronic supplementary material:**

The online version of this article (doi:10.1186/s12884-015-0664-3) contains supplementary material, which is available to authorized users.

## Background

A growing body of literature suggests that women who undergo treatment for cervical disease are at increased risk of preterm birth, particularly when the length of the tissue excised is greater than or equal to 10 mm [1–8]. In England, the absolute risk of preterm birth among treated women increased with the length of excision from 7.5 % (with length <10 mm) to 18 % in those with excisions greater than 20 mm [8].

Although the risk of preterm birth following excision is often cited as a reason for not offering cervical screening to young women, we are not aware of any papers formally calculating the risk associated with screening per se. Further whilst preterm births (as a proportion of all births) have been increasing in many countries [9, 10] it is unclear what proportion may be attributable to prior treatment for cervical disease.

In this study, we estimate the number of preterm births among women with cervical excisional treatments, accounting for age at excision and length of excision, in order to estimate the number and proportion of preterm births in England attributable to cervical tissue excision of 10 mm deep or more.

## Methods

### Subjects

**Fig. 1:**
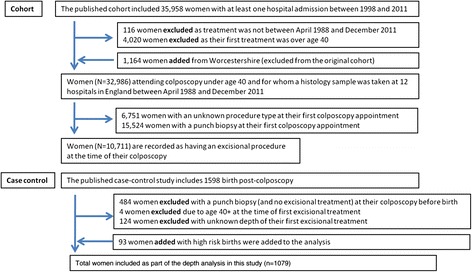
Inclusions and exclusions from the study

Women with cervical histology between April 1988 and December 2011 were identified from clinical records in 13 National Health Service (NHS) hospitals (see acknowledgments). The women were linked to hospital obstetric records between April 1998 and March 2011 for the whole of England using their NHS number (a unique identifier) and date of birth by HES (Hospital Episode Statistics), a data warehouse containing details of all admissions to NHS hospitals in England [11]. Minimal details were obtained for this cohort: date of first and last attendance to colposcopy and whether they had a punch biopsy or excisional treatment at these appointments. Excisional treatments were defined as: LLETZ, laser excision, non specified cone excision or knife cone biopsy. Details of women included in this study in comparison to published manuscripts [8, 12] are presented in Fig. 1.

From this cohort we identified women who had at least one singleton live birth with a gestational age of 20–42 weeks. We then selected the first singleton preterm birth (gestational age of 20–36 completed weeks) in each woman and frequency matched these to singleton term births (38–42 completed weeks) in women with no preterm births. Births at 37 weeks gestational age were excluded when performing the matching to allow a clear divide between term and preterm births. Full details on the study design have been published previously [8, 12]. From HES records we obtained detailed obstetric information. Hospitals entered colposcopy details into a study database and additionally submitted anonymised pathology reports to Barts Health NHS Trust. Pathology reports were entered into the study database by two trained individuals (AP and TP) to ensure measurements were entered in a standardised way, facilitating the identification of the length, thickness and circumference of specimens. Participating pathology departments confirmed that usual practice was to record the length of the specimen first on the pathology reports. Individuals searching for and coding colposcopy information were blind to the case–control status of the women.

Women were not eligible for the case–control study if detailed colposcopy information was not available, if colposcopy records were known to be incomplete, if the only pathology sample reported was non-cervical or if the woman was diagnosed with cervical cancer at any time. We also excluded one woman who was recorded as being sterilized whilst pregnant. Details of women excluded from the case–control study have been published previously [8]. Here we further exclude the following women: those attending colposcopy but not receiving excisional treatment, those who had their first treatment aged 40 and over and those with unknown length of their only excisional treatment (see Fig. 1). In our previous publication births as a result of a high risk pregnancy (defined elsewhere [8]) were excluded; however they are included here to determine the age at first excisional treatment, to ensure the sample is representative of the general population.

### Ethical approval

This study was approved by the Brompton, Harefield, and NHLI research ethics committee, Charing Cross Hospital, London (study reference number: 09/h0708/65). Since this was a retrospective linkage study using routinely recorded data, informed consent was not required by the research ethics committee. All data received by the researchers were anonymous.

#### Statistical methods

To assess the concordance between dates at first colposcopy procedure recorded in the cohort and the case–control study we selected all women recorded in the case–control study as having an excisional treatment as their first colposcopic procedure. We used a paired sample *t*-test to compare the age at first colposcopic appointment in the case–control study and the cohort study.

Length of excision was defined as the distance from the distal or external margin to the proximal or internal margin of the excised specimen, as defined by the IFCPC nomenclature [13]. The length of treatment was coded as per the pre-specified statistical analysis plan: excision with a length of <10 mm (small), 10–14 mm (medium), 15–19 mm (large) and ≥20 mm (very large). When the excision was piecemeal, the largest fragment length was used. For women with more than one excisional treatment (*n* = 106), if the treatments were within a year (*n* = 40), the lengths were summed, as is it is extremely unlikely there would be a birth between the two treatments. Length of treatments that were more than a year apart (*n* = 66) were summed at the time of the later treatment.

The distribution of length of excisional treatment was calculated using all available data for each age group (<20, 20–24, 25–29, 30–34, 35–39) in the case–control study, weighted using the inverse probability of selection into the case–control study to reflect the distribution of cases and eligible controls in the cohort. These data were combined with cohort data on the number of women in each age group who underwent an excisional treatment between April 1988 and December 2011. Using these data we estimate by age group the number of women in the cohort who had an excisional treatment in each length category. We assume that single excisions with unknown length have the same distribution as those with known length in the same age group. Women with an unknown length of first treatment but known length of second treatment within a year were assigned the median length observed among women who had multiple treatments within a year for the unknown length. For women with multiple treatments which were at least a year apart, if either treatment had an unknown length they were proportionally assigned to the length categories of treated women who had multiple treatments within the same age group.

Using age-specific fertility rates for England from 2010, [14] we estimated the expected number of future births per woman in 5-year age groups. We have previously shown that the increased risk of preterm birth remains for all future births, not just the first birth after colposcopy [15]. Therefore we applied published absolute risks [8] of preterm (20–36 gestational weeks) and very preterm (20–31 gestational weeks) births for each length category to the expected number of future births for women in each age group. The absolute risks of a preterm birth were 7.5 % following small excisions, 9.6 % following medium, 15.3 % following large and 18.0 % following very large excisions (for very preterm they were 2.0 %, 2.3 %, 3.6 % and 6.4 % respectively) [8]. Women with multiple excisional treatments not within a year of each other were assigned the absolute risk appropriate for their first treatment length from the age at their first excisional treatment to their second treatment, and the absolute risk associated with the two treatment lengths summed from the age at the second treatment. The expected number of preterm births was then estimated assuming that all treatments were small, to allow estimation of the excess risk of preterm birth caused by deeper excisions.

To estimate the number of preterm births associated with excisional treatments in England, it was necessary to estimate the number of women per year who have excisional treatments. For this we sourced the number of tests taken and the result of these tests from routinely published screening data for England in the financial year 2013/14 [16]. We then used previously published methodology [17] to estimate (from the published data) the total number of women aged 20–39 who had an excisional treatment in financial year 2013/14. Full details on how this was estimated can be found in Additional file 1, and the results in Additional file 2: Tables S1 and Additional file 3: Table S2.

Analyses were carried out in STATA 12 (StataCorp. College Station, Texas, USA).

## Results

Information was available on 32,986 women with a histological sample taken under age 40 at colposcopy between April 1988 and December 2011. From this cohort, around two-fifths (*n* = 10,711) of the women with known treatment at their first colposcopy (*N* = 26,235) were recorded as having an excisional procedure at their first attendance to colposcopy. Most of these women (53.9 %) were aged 25–34 at their first treatment (Table 1). From the case–control study 1079 women treated at colposcopy were eligible for this analysis; of these 894 were treated on their first attendance to colposcopy. This subset of women (*n* = 894) tended to be younger than women in the cohort, with 70.2 % treated age 20–29. This was expected, as women attending colposcopy at a younger age are more likely to go on to have a birth, and therefore be eligible for the case–control study. Comparison of the data from the cohort with that from the case–control study (for these 894 women) showed that the age at first treatment agreed exactly for 49.2 % of women and was within a month for an additional 32.8 %. Only 4.3 % differed by over 6 months. There was no significant difference in the age distributions (paired *t*-test *p*-value = 0.2319).

**Table 1:** Age among women whose first attendance to colposcopy resulted in excisional treatment. Comparison of cohort and case–control data

Age at treatment	Case–control study		Treated at first colposcopy in cohort among those included in the case–control study		Cohort study	
	Treated at first colposcopy				Treated at first colposcopy	
	N	%	N	%	N	%
<20	20	2.2	18	2.0	167	1.6
20–24	286	32.0	286	32.0	2355	22.0
25–29	342	38.3	340	38.0	3417	31.9
30–34	181	20.2	191	21.4	2907	27.1
35–39	65	7.3	59	6.6	1865	17.4
Total	894	100 %	894	100 %	10711	100 %

Estimated (weighted) age at first excisional treatment and proportion in each excisional length category among women in the cohort study is shown in Table 2. Overall the majority of first excisional treatments at colposcopy were small (47.5 %) or medium (39.1 %), 9.5 % were large and 4.1 % were very large excisions. Only 4.0 % of women treated before giving birth are estimated to have had a second excisional treatment more than a year after the first. The estimated number of births following excisional treatment by age group and the estimated excess preterm births due to treatment can be seen in Table 3. Thus based on our cohort of 10,711 treated women and the distribution of treatment lengths observed in the case control study we estimate an excess of 240 preterm births (including 57 very preterm) or 2.2 (including 0.5 very preterm) per 100 women treated.

**Table 2:** Estimated (weighted) age at first excisional treatment by length of tissue removed among women in the cohort study

	<10 mm		10–14 mm		15–19 mm		20 + mm		Total
	N	%	N	%	N	%	N	%	
<20	42.1	36.1	64.2	54.9	9.5	8.2	1.0	0.9	116.9
20–24	836.9	52.5	535.7	33.6	154.5	9.7	68.2	4.3	1595.3
25–29	957.8	42.0	974.4	42.8	238.3	10.5	108.4	4.8	2278.8
30–34	510.3	49.8	424.0	41.4	62.7	6.1	27.1	2.6	1024.0
35–39	140.5	58.5	56.7	23.6	32.6	13.6	10.5	4.4	240.3
Total	2487.6	47.3	2054.9	39.1	497.6	9.5	215.2	4.1	5255.3

**Table 3:** Estimated preterm births due to excisional treatment of the cervical transformation zone

			Estimated births after first treatment (N)	Preterm births			Very preterm births		
				(<37 gestational weeks)			(20–31 gestational weeks)		
Age at first treatment	Women with excisional treatment			Estimated births taking into account length distribution*	Estimated births assuming all excisions ≥10 mm were small	Excess births due to treatments	Estimated births taking into account length distribution	Estimated births assuming all excisions ≥10 mm were small	Excess births due to treatments
	N	%							
<20 single excision	167	1.6	323	31	24	7	8	6	1
20–24 single excision	2355	22.0	4043	395	303	91	104	81	23
25–29 single excision	3417	31.9	4332	428	325	103	111	87	24
30–34 single excision	2907	27.1	1929	176	145	31	45	39	7
35–39 single excision	1865	17.4	343	33	26	7	9	7	2
Total	10711	100 %	10970	1063	823	240	276	219	57

At a population level (for England) we estimate that 39,101 women aged 20–39 would be treated each year (Table 4, and Additional file 1). We estimate that these treatments will lead to an excess of 840 preterm births (including 196 very preterm) in England each year among women with excisional treatments with a length of ≥10 mm.

**Table 4:** Estimated number of women referred and treated at colposcopy due to cervical intraepithelial neoplasia grade 2 (CIN2+) or worse diagnosis and the resulting excess preterm births each year in England

	N screening tests:	N referred to colposcopy:	N referred with mild	N referred with mod+	N with CIN2+:	Estimated excess number of future preterm births		Estimated excess number of future very preterm births	
						per woman treated	in women treated	per woman treated	in women treated
20–24	46,050	4,668	2,852	1,816	2037	0.04	79	0.01	20
25–29	566,057	46,468	27535	18933	20844	0.03	630	0.01	149
30–34	497,109	24,640	16178	8462	10004	0.01	108	0.00	22
35–39	446,807	16,086	11036	5050	6216	0.00	23	0.00	6
total	1,556,023	91,861	57,600	34,261	39,101		840		196

## Discussion

Based on the observed age at first excisional treatment at colposcopy in women under age 40, the observed length of tissue removed, age specific fertility rates and the length specific absolute risks of preterm birth we have estimated that treatment for cervical disease leads to 2.2 preterm (0.5 very preterm) births per 100 women treated under age 40. At a national level this corresponds to 840 preterm births per year in England.

### Strengths and limitations

Our results are based on the assumption that the association between preterm birth and treatment for cervical disease is causal. However lifestyle factors (such as smoking) were not adjusted for when estimating the risks used in this study, thus it is possible that residual confounding remains.

The distribution of age at first excisional treatment for women in England is extrapolated from that observed in our study and may not be representative of all women currently being treated in England. For example, women aged 20–24 are no longer invited for cervical screening in England, so we expect the number of preterm births due to excisional treatment among women in this age group to be lower than the number estimated here. Only 2.7 % of all treatments in the study were carried out before age 25, so the impact on the number of preterm birth is likely to be small.

Although the NHS hospital trusts providing data to this study were self selected they did not differ (on the basis of published data) from other colposcopy clinics in England [8]. We therefore think it is justified to assume that the observed distribution of excised tissue length can be extrapolated to all women in England.

Due to the lack of routinely reported data, it was necessary to estimate the number of women treated each year at colposcopy in England. Our estimate (*n* = 39,101) is based on routinely reported cytology and histology data, however it is worth noting that the results are similar to twice the number of CIN3 diagnoses in women under age 40 (*n* = 40,750) [18]. Diagnoses of CIN2 are not routinely recorded or reported, but we think it sensible to assume that the number diagnosed is similar to that of CIN3 and that most of these women would have an excisional treatment as a result. This gives us confidence that our estimate of women treated at colposcopy each year is robust.

We have made assumptions regarding the length of excision among samples where the measurements are unknown, including those with multiple treatments. The risk in those with unknown length was found to be similar to those with medium excisions [8] suggesting that factors other than length (such as piecemeal excisions) are responsible for missing values.

We have applied absolute risks of preterm birth to all future births, not just the first following excisional treatment. We believe this is justified since we have shown that an increased risk of preterm birth remains for all future births, not just the first birth following treatment [15].

We have estimated the excess number of preterm birth attributable to excisional by comparing the observed length distribution in our study to a scenario where all excisions have a length of less than 10 mm (equivalent to the baseline risk in those attending colposcopy). We are not suggesting that clinical practice should change to force all excisions to be small, we are purely estimating the excess risk attributable to excisions with a length greater than 10 mm.

### Interpretation

Relative risks of preterm birth following large (17 + mm) treatments compared to small treatments (≤10 mm) have been reported to be between 1.74 [7] and 1.79 [3] in Scandinavian populations and up to 2.4 in an English population [8]. A meta-analysis [5] comparing untreated women to those with excisions deeper than 10 mm found the relative risk of preterm delivery to be 2.61 (95 % CI 1.28–5.34), though a study from Belgium [19] found even higher risks (RR = 4.55, 95 % CI:1.32–15.65). The study in England [8] found that the absolute risk of preterm birth among those who receive a diagnostic biopsy (7.2 %, 95 % CI: 5.9 %–8.5 %) is similar to that of women with small treatments (7.5 %, 95 % CI: 6.0 %–8.9 %). Nevertheless women attending colposcopy following an abnormal cytology result are at a higher risk of preterm birth than the general population in England, where the preterm rate in England is 6.7 % [8]. This is most likely due to the shared risk factors for preterm delivery and cervical disease.

An excess of 2.2 preterm births per 100 women treated may not seem like much, however the average number of births in England between 2000 and 2009 was 510,660 each year and the preterm rate during this period was 6.7 % (or 33,168 births) [12]. Extrapolating the results observed in this study to the whole of England, we would expect 840 preterm births a year (equivalent to 2.5 % of preterm births in England) to be due to excisional treatments of length ≥10 mm.

The risk of preterm birth following treatment for cervical disease can be considered a harm of cervical cancer screening. Nevertheless it needs to be considered against the 20,375 women under age 40 diagnosed and treated for carcinoma in situ of the cervix uteri (CIN3) in 2012 [18], many of whom would have gone on to develop cervical cancer without intervention.

Colposcopists will need to carefully consider whether the benefits of a small excision outweigh the potential risk of leaving diseased tissue on the cervix (i.e. positive margins), risking the need for further treatment. Patients who need a large excision of the cervix should be informed about the risk of preterm birth. Close obstetric monitoring is warranted for women who undergo large excisional treatment and subsequently become pregnant, particularly since recently published evidence suggests that preterm birth in high risk pregnancies can be substantially reduced by measuring fibronectin levels and assessing cervical length [20]. The extra costs of close obstetric monitoring of these women will easily be offset by the saving made in the reduction of preterm births, which cost on average 1.47 % more than term babies up to age 18 [21].

## Conclusion

We estimate that 840 or 2.5 % of preterm births in England each year are due to excisional treatments of 10 mm or more. Clinicians (and in particular obstetricians) need to be aware of whether women in their care have undergone treatment of the cervical transformation zone. Those who have must undergo close monitoring and further investigations with the aim of reducing the number of preterm births among these women.

## Additional files

Additional file 1:
**Data and methodology to estimate the number of women receiving excisional treatment for cervical disease each year in England.** (DOC 24 kb) Additional file 2: Table S1. Routinely published screening data from England in the financial year 2013/14. (DOC 53 kb) Additional file 3: Table S2. Outcomes of referrals to colposcopy in England in the financial year 2013/14. (DOC 33 kb)
