# Supplementary material for: How many preterm births in England are due to excision of the cervical transformation zone? Nested case control study
Source: BMC Pregnancy Childbirth. 2015 Sep 29;15:232. doi: 10.1186/s12884-015-0664-3 (PMC4588250; doi:10.1186/s12884-015-0664-3)
Supplement: Additional file 1: — Data and methodology to estimate the number of women receiving excisional treatment for cervical disease each year in England. (DOC 24 kb) [file 12884_2015_664_MOESM1_ESM.doc]

**Additional file 1.** Data and methodology to estimate the number of women receiving excisional treatment for cervical disease each year in England.

To estimate the number of preterm births associated with excisional treatments in England, we first estimated the total number of women aged 20-39 who had an excisional treatment in financial year 2013/14. We used routinely published screening data from England in the financial year 2013/14^1^ for the number of tests taken and the result of these tests (see Table S1 and Table S2 below). Methods on how to estimate the number of women treated each year using routinely published screening data from England have been published previously. (ref)

Routinely published cervical screening statistics on the numbers of referrals to colposcopy are not reported by age. To estimate referrals within the age groups we assumed that all women with a moderate or worse cytology test result and a certain proportion (independent of age) of women with a borderline or mild test result are referred to colposcopy. This proportion is estimated from subtracting the number of moderate or worse dyskaryosis results from the number of referrals to colposcopy, and expressing this as a proportion of all borderline/low-grade cytology results.

As there are no good data on the number of women who undergo excisional treatments each year, we approximated the number treated by the number with CIN2 or worse on histology. Since there are no data on histology by age, we assumed that the positive predictive value of a particular cytology result for CIN2+ was the same at all ages. The predictive values are given separately for women referred following a low-grade result (inadequate, borderline and mild) and women with a potentially significant abnormality (moderate or worse cytology). These numbers were estimated in 5-year age groups. The estimated excess number of preterm births per woman treated in each 5-year age group was then multiplied by the number of women treated in the 5-year age group to estimate the total number of excess preterm births from women treated aged 20-39 in financial year 2013/14.

**References:**

1. Cervical Screening Programme, England, Statistics for 2013-14. [database on the Internet] 2014. Available from: <http://www.hscic.gov.uk/catalogue/PUB15968/cerv-scre-prog-eng-2013-14-rep.pdf>.

2. Landy R, Birke H, Castanon A, Sasieni P. Benefits and harms of cervical screening from age 20 years compared with screening from age 25 years. British journal of cancer, 2014;110:1841-6.
