# Supplementary material for: How many preterm births in England are due to excision of the cervical transformation zone? Nested case control study
Source: BMC Pregnancy Childbirth. 2015 Sep 29;15:232. doi: 10.1186/s12884-015-0664-3 (PMC4588250; doi:10.1186/s12884-015-0664-3)
Supplement: Additional file 2: Table S1. — Routinely published screening data from England in the financial year 2013/14. (DOC 53 kb) [file 12884_2015_664_MOESM2_ESM.doc]

| **Table S1**: Cross sectional screening data from English routine statistics | | | |  |
| --- | --- | --- | --- | --- |
|  |  | **England** | |  |
|  | Age group | Number | % |  |
| Population^(1)^ | 20-24 | 1,916,000 |  |  |
|  | 25-29 | 2,046,400 |  |  |
|  | 30-34 | 2,018,300 |  |  |
|  | 35-39 | 1,793,800 |  |  |
|  | All ages | 28,389,400 |  |  |
| Proportion of women screened in year | 20-24 |  | 2.4% |  |
|  | 25-29 |  | 27.7% |  |
|  | 30-34 |  | 24.6% |  |
|  | 35-39 |  | 24.9% |  |
|  | All ages |  | 11.5% |  |
| Screening tests^(2)^ | 20-24 | 46,050 |  |  |
|  | 25-29 | 566,057 |  |  |
|  | 30-34 | 497,109 |  |  |
|  | 35-39 | 446,807 |  |  |
|  | All ages | 3,259,309 |  |  |
| Negative test results^(2)^ | 20-24 | 38,653 | 83.9% |  |
|  | 25-29 | 493,653 | 87.2% |  |
|  | 30-34 | 456,986 | 91.9% |  |
|  | 35-39 | 420,160 | 94.0% |  |
|  | All ages | 3,040,116 | 93.3% |  |
| Non-negative test results^(2)^ | 20-24 | 7,397 | 16.1% |  |
|  | 25-29 | 72,404 | 12.8% |  |
|  | 30-34 | 40,123 | 8.1% |  |
|  | 35-39 | 26,647 | 6.0% |  |
|  | All ages | 219,193 | 6.7% |  |
| Borderline/Low-grade Dyskaryosis^(2)^ | 20-24 | 5,581 | 12.1% |  |
|  | 25-29 | 53,886 | 9.5% |  |
|  | 30-34 | 31,661 | 6.4% |  |
|  | 35-39 | 21,597 | 4.8% |  |
|  | All ages | 175,548 | 5.4% |  |
| Moderate Dyskaryosis | 20-24 | 1,816 | 3.9% |  |
| or worse^(2)^ | 25-29 | 18,933 | 3.3% |  |
|  | 30-34 | 8,462 | 1.7% |  |
|  | 35-39 | 5,050 | 1.1% |  |
|  | All ages | 43,645 | 1.3% |  |
| Referred to colposcopy^(3)^ | All ages | 133,346 |  |  |
| ^(1)^ Source: Cervical Screening Programme 2013/14 - The NHS Information Centre, England (Table 2) | | | | |
| ^(2)^ Source: Cervical Screening Programme 2013/14 - The NHS Information Centre, England (Table 8); Excluding the number of inadequate test results; Most severe result in year | | | | |
| ^(3)^ Source: Cervical Screening Programme 2013/14 - The NHS Information Centre, England (Table 20); Referral indication borderline, mild, moderate and worse test result | | | | |
| ^*^We estimate that all women with moderate or worse dyskaryosis are referred to colposcopy and 51.5% of those with borderline/low-grade | | | | |
