# Supplementary material for: How many preterm births in England are due to excision of the cervical transformation zone? Nested case control study
Source: BMC Pregnancy Childbirth. 2015 Sep 29;15:232. doi: 10.1186/s12884-015-0664-3 (PMC4588250; doi:10.1186/s12884-015-0664-3)
Supplement: Additional file 3: Table S2. — Outcomes of referrals to colposcopy in England in the financial year 2013/14. (DOC 33 kb) [file 12884_2015_664_MOESM3_ESM.doc]

| **Table S2**: Outcome of referrals ^(1)^ | | | | |  |
| --- | --- | --- | --- | --- | --- |
|  | **England** | |  |  | |
|  | Inadequate/ Borderline/ Mild test results | Moderate worse test results^(2)^ |  |  | |
| Number of women | 86,166 | 36,137 |  |  | |
| Cervical Cancer | 0.1% | 2.7% |  |  | |
| Non-Cervical Cancer | 0.0% | 0.5% |  |  | |
| CIN3 and AIS | 6.9% | 57.1% |  |  | |
| CIN2 | 10.7% | 23.9% |  |  | |
| ≤CIN1/HPV | 52.3% | 13.6% |  |  | |
| Colposcopy NAD/ Inadequate biopsy | 29.9% | 2.2% |  |  | |
| All high-grade^(3)^ | 17.8% | 84.2% |  |  | |
| ^(1)^ Source: Cervical Screening Programme 2013/14 – The NHS Information Centre, England (Table 18) | | | | |  |
| ^(2)^ Include Moderate/ Severe Dyskaryosis, Severe/?Invasive Carcinoma and ?Glandular Neoplasia | | | | |  |
| ^(3)^ Including cervical cancer, non-cervical cancer, CIN3 and Adenocarcinoma in situ and CIN2 | | | | |  |
